# Secondary Metabolites Control the Associated Bacterial Communities of Saprophytic Basidiomycotina Fungi

**DOI:** 10.1264/jsme2.ME14139

**Published:** 2015-04-23

**Authors:** Maira Peres de Carvalho, Patrick Türck, Wolf-Rainer Abraham

**Affiliations:** 1 Helmholtz Centre for Infection Research, Chemical MicrobiologyInhoffenstrasse 7, 38124, BraunschweigGermany; 2 Universidade Federal do Rio Grande do Sul, Laboratório de Neurobiologia CelularAv. Bento Gonçalves, 9500, Porto AlegreBrazil

**Keywords:** Basidiomycotina, biofilm, fruiting bodies, SSCP

## Abstract

Fungi grow under humid conditions and are, therefore, prone to biofilm infections. A 16S rRNA fingerprint analysis was performed on 49 sporocarps of Basidiomycotina in order to determine whether they are able to control these biofilms. Ninety-five bacterial phylotypes, comprising 4 phyla and 10 families, were identified. While ectomycorrhizal fungi harbored the highest bacterial diversity, saprophytic fungi showed little or no association with bacteria. Seven fungal species were screened for antimicrobial and antibiofilm activities. Biofilm formation and bacterial growth was inhibited by extracts obtained from saprophytic fungi, which confirmed the hypothesis that many fungi modulate biofilm colonization on their sporocarps.

Fungi are a large group of organisms that colonize a multitude of habitats. Mycelia and fruiting bodies contribute differently to the life cycle of a fungus, in which vegetative mycelia are responsible for substrata colonization and nutrient uptake and the fruiting bodies, typically short-lived forms, are involved in reproduction. Bacteria and fungi have co-existed for billions of years, sharing the same ecological niches and often depending on each other. They have established complex relationships, which can be either beneficial or deleterious (1, 16). To modulate those interactions with their friendly and unfriendly counterparts, fungi rely on their chemical self-defense mechanisms, which confer protection against environmental threats (2, 11, 16, 17).

A broad range of molecules, varying from simple to highly complex structures, has been isolated as fungal products. These metabolites are biosynthesized for specialized physiological, social, or protective reasons, and confer environmental advantages to the producer. Since penicillin’s discovery, the importance of fungal metabolites in the search for new drugs became evident. However, no screening approaches that consider fungal-bacteria ecological aspects have yet been performed. Previous studies on these interactions have been restricted to culturable bacteria isolated from the fruiting bodies of symbiotic and economically important species (9, 12, 19, 21).

In the present study, the compositions of bacterial communities associated with 49 Basidiomycotina fruiting bodies, both saprophytic and ectomycorrhizal, were analyzed using a DNA fingerprint method (see Table S1 for fungal species, collection venues, and GenBank sequences accession numbers). In addition, some of those fungal species were used to screen for antimicrobial and antibiofilm compounds.

A single-strand conformation polymorphism (SSCP) analysis was employed to evaluate fungal-associated bacterial communities. The fungi collected were identified according to their morphological characteristics, and included one individual of each species submitted to ITS region sequencing. In the SSCP analysis, DNA was extracted from a mushroom pileus skin fragment using FastDNA Spin kit for Soil (MP Biomedicals) and quantified with a NanoDrop spectrophotometer. PCR reactions were performed using the primer set 27F-521R, generating a fragment of approximately 500 bp (15, 22). Although a sufficient amount of DNA was yielded, 19 fungal strains did not show bacterial DNA amplification. The SSCP fingerprints of the remaining 30 samples allowed for the quantification of 118 bacterial phylotypes. The highest bacterial diversity was associated with the ectomycorrhizal fungi *Boletus* spp. and *Russula emetica*. Remarkably, only 3 out of the 17 *Coprinus comatus* specimens harbored bacteria (Fig. 1).

To identify the members of these communities, the identities of the 16S rRNA consensus sequences obtained were confirmed using the Seqmatch feature available at the RDP-II database (5). Multiple sequence alignments were performed using the program Muscle, provided in the software SeaView 4.0 (8). MEGA 5.0 was used to construct phylogenetic trees using the neighbor-joining method together with the Jukes–Cantor model and pairwise deletion of gaps to calculate the evolutionary distance (18). A total of 1,000 bootstrap replications were executed to test for branch robustness. DECIPHER was employed to search for chimeras (23) and the sequences were deposited in GenBank.

SSCP fingerprinting led to the identification of 95 phylotypes, grouped into 4 phyla and 10 families: 56 *Gammaproteobacteria* (34 *Enterobactereaceae*, 20 *Pseudomonadaceae*, 1 *Moraxellaceae*, and 1 *Xanthomonadaceae*), 21 *Alphaproteobacteria* (15 *Rhizobiaceae*, 5 *Bradyrhizobiaceae*, and 1 *Acetobactereaceae*), 13 *Betaproteobacteria* (3 *Burkholderiaceae* and 10 *Oxalobactereaceae*) and 5 *Bacteroidetes*, all belonging to *Flavobacteraceae* (Fig. S1). The sequences of the 23 remaining phylotypes (2 OTU1, 1 OTU2, 9 OTU3, 10 OTU4, and 1 OTU5) displayed a low resolution, thereby preventing their phylogenetic placement. To investigate specific bacterial-fungal interactions, PRIMER 6 (v.6.1.6, PRIMER-E, Plymouth Marine Laboratory, UK) was used to perform a non-parametric multivariate statistical analysis (3, 4). A binary matrix was generated and subjected to a modified Bray–Curtis similarity measure in order to compare the presence/absence of each of the 118 bacterial phylotypes distributed over the 30 mushroom samples (13). The results are displayed as a dendrogram (Fig. S2).

Fifteen phylotypes, identified as *Rhanella* spp., were mostly associated with *Boletus* spp., while 20 rRNA sequences, identified as *Pseudomonas* spp., showed a broad distribution between the analyzed fungal genera. Interestingly, the phylotypes at *Russula emetica* fruiting bodies showed a very close association with *Rhizobiaceae*. Regarding these individuals, 15 bacterial phylotypes were detected, 13 of which belonged to *Rhizobiaceae* and 2 to *Flavobacterium* sp. Furthermore, communities associated with *Boletus* spp. possessed a high prevalence of *Enterobactereaceae*.

The term fungiphile was introduced to characterize bacteria that are often detected in association with fungi. *Pseudomonas* spp. and *Rhanella* spp. were identified from several mycosphere samples using DGGE (21). Previous studies on bacterial communities associated with hyphosphere soil and the fruiting body surfaces and internal tissues of ectomycorrhizal species, using culturable methods, showed a prevalence of Gram-negative bacteria (6, 24). A comparison of arbuscular mycorrhizal fungi-associated bacteria, considering both attached and non-attached communities, revealed that Gram-negatives were better hyphae colonizers (14). Warmink and van Elsas (20, 21) discussed the involvement of the type-III secretion system (TTSS) on the bacterial cells binding to *Laccaria proxima* hyphae, particularly *Pseudomonas* spp. TTSS are virulence-associated systems that are widespread among symbiotic and pathogenic *Proteobacteria*, and are involved in interactions between bacteria and their respective hosts (7).

A preliminary screening for antimicrobial and antibiofilm activities was performed employing *Boletus aestivalis*, *Coprinus comatus*, *Coprinopsis picaceae*, *Macrolepiota fuliginosa*, *Macrolepiota procera*, and *Russula emetica* fruiting bodies. Fresh fruiting bodies were macerated and extracted with methanol overnight at 4°C in the dark. The solvent was removed under a vacuum at 37°C. *Bacillus cereus* DSM 626, *Escherichia coli* DSM 498, *Pseudomonas aeruginosa* PA 14, and *Staphylococcus aureus* DSM 1104 were used to assess the Minimal inhibitory concentrations (MIC) of the crude extracts. Bacteria were inoculated in LB medium in 96-well microtiter plates and the extracts were added in serial dilutions, ranging from 7.8 μg mL^−1^ to 1,000 μg mL^−1^. Plates were incubated at 37°C over 20 to 24 h. The minimal biofilm inhibitory concentrations (MBIC) of *P. aeruginosa* and *S. aureus* were also assessed. Both strains were allowed to grow on 96-well plates at 37°C in LB for *P. aeruginosa* and casein-soja-peptone (CASO) broth for *S. aureus*, together with the mushroom crude extracts (same concentration range as above). *P. aeruginosa* plates were washed with PBS buffer after a 24 h incubation and *S. aureus* plates after a 16 to 20 h incubation. Crystal violet was used to stain the microtiter plates (10). MIC and MBIC values are shown in Table 1. The organic extracts obtained from *C. comatus*, *L. sulphureus*, *M. procera*, and *M. fuliginosa* showed either antimicrobial or antibiofilm activities. Most of these extracts lacked bacterial communities attached to their pileus. On the other hand, *B. aestivalis*, *C. picaceae*, and *R. emetica* extracts did not show any of these activities, and interestingly harbored the highest bacterial diversity among the evaluated samples. In summary, the results of the present study indicate that an appropriate level of knowledge on fungi and bacteria interactions will be beneficial for determining approaches to screen for compounds exhibiting biological activities.

Nucleotide sequence data have been submitted to the GenBank database under the accession numbers KM522713–KM522770.

## Supplementary Information



## Figures and Tables

**Fig. 1:**
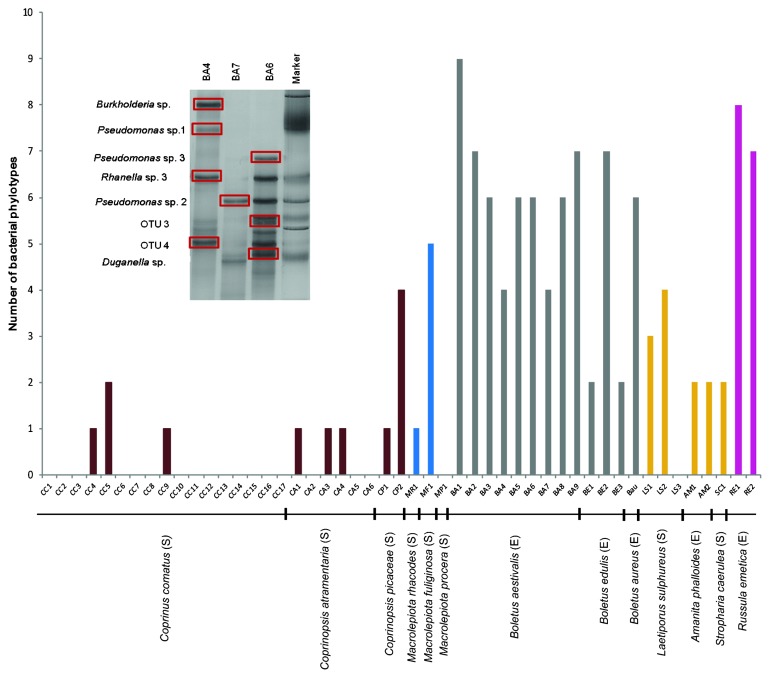
Number of bacterial phylotypes detected on 49 mushrooms hats. The details of a SSCP gel are shown in the insert. (E)–Ectomycorrhizal and (S)–Saprophytic.

**Table 1:** Minimal inhibitory concentrations (MIC) and minimal biofilm inhibitory concentrations (MBIC) of crude extracts obtained from mushroom fruiting bodies.

Fungal extract^a^	Bacterial strains					

	*B. cereus*	*E. coli*	*P. aeruginosa*		*S. aureus*	
			
	MIC^b^	MIC^b^	MIC^b^	MBIC^b^	MIC^b^	MBIC^b^
*B. aestivalis* (E)	—	—	—	—	—	—
*C. comatus* (S)	500	500	500	—	250	
*C. picaceae* (S)	—	—	—	—	—	—
*L. sulphureus* (S)	—	—	—	—	—	125
*M. fuliginosa* (S)	500	—	—	—	500	125
*M. procera* (S)	250	—	—	—	250	62.5
*R. emetica* (E)	—	—	—	—	—	—

a (E)–Ectomycorrhizal species; (S)–Saprophytic species.

b MICs and MBICs are expressed in μg mL^−1^.
