# Supplementary material for: Secondary Metabolites Control the Associated Bacterial Communities of Saprophytic Basidiomycotina Fungi
Source: Microbes Environ. 2015 Apr 23;30(2):196–8. doi: 10.1264/jsme2.ME14139 (PMC4462932; doi:10.1264/jsme2.ME14139)
Supplement: Supplementary file 1 [file 30_196_s1.pdf]

Table S1. Fungal species used in the SSCP analysis, together with their respective abbreviation, collection venue (BS - Braunschweig; HZI - Helmholtz Center for Infection Research; GÖ – Göttingen; KÖ – Köris; DRÜ – Drübeck) and year, as well as GenBank accession numbers.

Fungal Species	Abbreviation	Collection venue/year	GenBank accession number
Saprophytic			
Coprinus comatus	CC1	BS – HZI/2009	KM522717
C. comatus	CC2	BS – HZI/2010	
C. comatus	CC3	BS – Bürgerpark/2010	
C. comatus	CC4	BS – Bürgerpark/2011	
C. comatus	CC5	BS – Bürgerpark/2011	
C. comatus	CC6	BS – HZI/2012	
C. comatus	CC7	BS – HZI/2012	
C. comatus	CC8	BS – HZI/2012	
C. comatus	CC9	BS – HZI/2012	
C. comatus	CC10	BS – HZI/2012	
C. comatus	CC11	GÖ/2010	
C. comatus	CC12	DRÜ/2012	
C. comatus	CC13	DRÜ /2012	
C. comatus	CC14	BS – HZI/2012	
C. comatus	CC15	BS – HZI/2012	
C. comatus	CC16	BS – HZI/2012	
C. comatus	CC17	BS – HZI/2012	
Coprinopsis atramentaria	CA1	BS – HZI/2012	Identified morphologically
C. atramentaria	CA2	BS – HZI/2012	
C. atramentaria	CA3	BS – HZI/2012	
C. atramentaria	CA4	BS - HZI/2012	
C. atramentaria	CA5	BS - HZI/2012	
C. atramentaria	CA6	BS - HZI/2012	
Coprinopsis picaceae	CP1	DRÜ /2012	Identified morphologically
C. picaceae	CP2	DRÜ /2012	
Laetiporus sulphureus	LS1	BS – Bürgerpark/2011	KM522720
L. sulphureus	LS2	BS - Bürgerpark/2011	
L. sulphureus	LS3	BS - Bürgerpark/2011	
Macrolepiota rhacodes	MR1	DRÜ /2012	
Macrolepiota fuliginosa	MF1	GÖ/2009	KM522719
Macrolepiota procera	MP1	KÖ/2011	KM522713
Stropharia caerulea	SC1	BS – HZI/2009	KM522718
Ectomycorrhizal			
Amanita phalloides	AM1	BS - HZI/2010	KM522716
A.phalloides	AM2	BS - HZI/2010	
Boletus aestivalis	BA1	BS – HZI/2009	KM522715
B. aestivalis	BA2	BS - HZI/2009	
B. aestivalis	BA3	BS - HZI/2010	
B. aestivalis	BA4	BS - HZI/2011	
B. aestivalis	BA5	GÖ /2010	
B. aestivalis	BA6	GÖ /2010	
B. aestivalis	BA7	GÖ /2011	
B. aestivalis	BA8	KÖ/2011	
B. aestivalis	BA9	KÖ/2011	
Boletus aureus	Bau	BS- HZI/2010	Identified morphologically
Boletus edulis	BE1	BS - HZI/2009	Identified morphologically
B. edulis	BE2	BS - HZI/2010	
B. edulis	BE3	GÖ/2009	
Russula emetica	RE1	BS - HZI/2009	Identified morphologically
R. emetica	RE2	BS – HZI/2011	

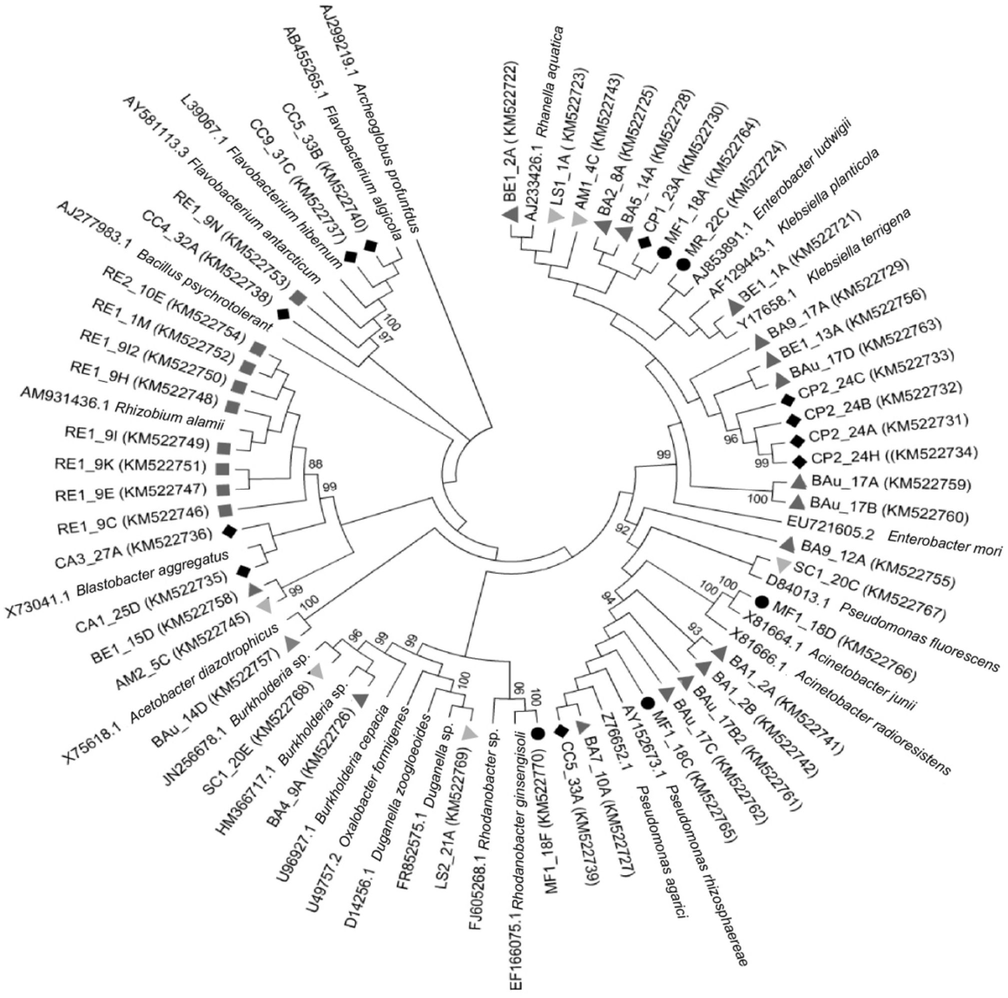

Fig. S1 - Neighbor joining tree of the 16S rRNA fragments sequences, obtained from SSCP fingerprint analysis from 30 mushrooms samples together with reference sequences. Evolutionary distances were computed using the Jukes-Cantor model. The sequence of *Archaeoglobus* served as outgroup. The Symbols square and triangle indicate ectomycorrhizal species; circle and diamond, saprophytic ones; inverted triangle comprises a mixed group.

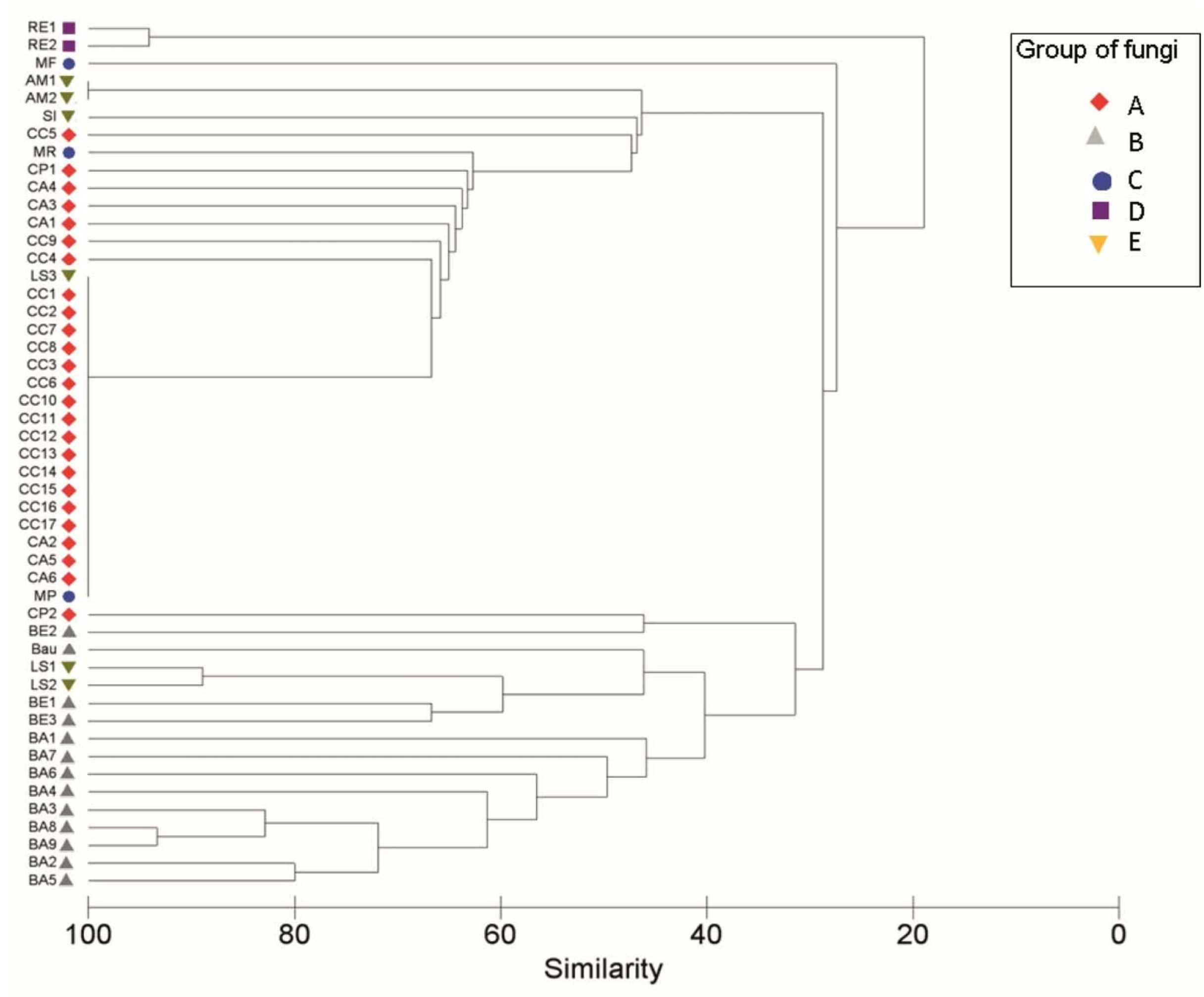

Fig. S2 - Fig. 2 - Dendrogram based on modified Bray Curtis similarity analysis, showing the differences between bacterial communities of 49 mushrooms. Samples were divided in 5 groups, in the following way: A) *Coprinus comatus* and *Coprinopsis* spp., B) *Boletus* spp., C) *Macrolepiota* spp., D) *Russula emetica,* E) mixed genera. Groups A and C comprise saprophytic fungi, while groups B and D the ectomycorrhizal ones. In group E, samples AM1 and AM2 are ectomycorrhizal and samples LS1 and LS2, saprophytic.
